# The recurrent Spike A222V mutation in SARS-CoV-2 enhances replication in primary deer lung cells

**DOI:** 10.1093/ve/veaf059

**Published:** 2025-08-05

**Authors:** Chelsea Cereghino, Kateland Tiller, Lin Kang, Pawel Michalak, James Weger-Lucarelli

**Affiliations:** Department of Biomedical Sciences and Pathobiology, 205 Duck Pond Drive, VA-MD College of Veterinary Medicine at Virginia Tech, Blacksburg, VA 24061, United States; Center for Emerging, Zoonotic, and Vector-borne Pathogens, Fralin Life Sciences Institute, Virginia Tech Corporate Research Center (CRC), Integrated Life Science Building (ILSB) 1981 Kraft Dr, Room 2036, Blacksburg, VA 24060, United States; Department of Biomedical Sciences and Pathobiology, 205 Duck Pond Drive, VA-MD College of Veterinary Medicine at Virginia Tech, Blacksburg, VA 24061, United States; Center for Emerging, Zoonotic, and Vector-borne Pathogens, Fralin Life Sciences Institute, Virginia Tech Corporate Research Center (CRC), Integrated Life Science Building (ILSB) 1981 Kraft Dr, Room 2036, Blacksburg, VA 24060, United States; Department of Biomedical Research, Edward Via College of Osteopathic Medicine, 4408 Bon Aire Drive, Monroe, LA 71203, United States; College of Pharmacy, University of Louisiana Monroe, 1800 Bienville Dr, Monroe, LA 71201, United States; Center for One Health Research, VA-MD College of Veterinary Medicine, 1410 Prices Ford Road, Blacksburg, VA 24060, United States; Department of Biomedical Research, Edward Via College of Osteopathic Medicine, 4408 Bon Aire Drive, Monroe, LA 71203, United States; Center for One Health Research, VA-MD College of Veterinary Medicine, 1410 Prices Ford Road, Blacksburg, VA 24060, United States; Institute of Evolution, University of Haifa, 199 Abba Khoushy Ave., Mount Carmel, Haifa, 3103301, Israel; Department of Biomedical Sciences and Pathobiology, 205 Duck Pond Drive, VA-MD College of Veterinary Medicine at Virginia Tech, Blacksburg, VA 24061, United States; Center for Emerging, Zoonotic, and Vector-borne Pathogens, Fralin Life Sciences Institute, Virginia Tech Corporate Research Center (CRC), Integrated Life Science Building (ILSB) 1981 Kraft Dr, Room 2036, Blacksburg, VA 24060, United States

**Keywords:** spike, A222V, SARS-CoV-2, host adaptation, white-tailed deer

## Abstract

Severe acute respiratory syndrome coronavirus 2 (SARS-CoV-2) infects humans and animals and is therefore a pathogen of grave concern within a One Health framework. Identifying animal-adaptive mutations is critical to preserving One Health, as these mutations could also lead to the persistence of SARS-CoV-2 in animal reservoirs with continual spillover to humans. Therefore, we sought to pair experimental evolution and epidemiological data to identify putative human- and animal-adaptive viral residues and determine their impact on replication-competent SARS-CoV-2 in both human and animal cells. We passaged SARS-CoV-2 in cells expressing human, dog, cat, mink, and white-tailed deer ACE2 and sequenced the passaged populations. In addition, we searched SARS-CoV-2 sequences for mutations following patterns of convergent evolution that were common to both human- and animal-derived SARS-CoV-2 sequences. We identified the epidemiologically relevant Spike A222V mutation from our passaging experiment in cells expressing cat ACE2, a mutation that has also arisen independently across eight lineages of SARS-CoV-2 from human- and animal-derived sequences. To assess its impact on replication in human and animal cells, we constructed SARS-CoV-2 Spike A222V in the Wuhan-Hu-1 backbone with Spike D614G; this virus replicated similarly to the WT SARS-CoV-2 in human lung epithelial cells. In contrast, SARS-CoV-2 Spike A222V demonstrated an advantage in replication in primary deer lung cells, which was not mediated by the deer ACE2 receptor. Infection *via* the human, dog, cat, and mink ACE2 receptor resulted in reduced replication of SARS-CoV-2 Spike A222V. Our experiments identified Spike A222V as a putatively deer-adaptive mutation. Future studies should assess Spike A222V’s relevance to transmission within deer and to other animal species in contact with deer.

## Introduction

Severe acute respiratory syndrome coronavirus 2 (SARS-CoV-2), the causative agent of coronavirus disease 2019 (COVID-19), remains a public health concern 5 years after the start of the pandemic. Cases of COVID-19 mortality in the USA in 2024 exceeded 47 000, and, as of February 2025, cumulative deaths in the USA since the beginning of the pandemic exceed 1.22 million (CDC 2020). Morbidity is not restricted to humans, as SARS-CoV-2 infects and causes disease in a variety of animals (Cui *et al*. 2022). A total of 775 outbreaks has been reported in animals to date (SARS-CoV-2 2023) with at least 2000 animal-derived SARS-CoV-2 genomes sequenced (Khare *et al*. 2021). A notable outbreak of SARS-CoV-2 in animals occurred in 2020 on mink farms in Denmark and the Netherlands. Detection of SARS-CoV-2 in mink was facilitated by observed respiratory symptoms in mink (Oreshkova *et al*. 2020) and ultimately led to the culling of large numbers of farmed mink (van Volksgezondheid and Sport 2020). COVID-19 symptoms have been reported in other species like domesticated cats (Garigliany *et al*. 2020), lions (McAloose *et al*. 2020), tigers (Grome *et al*. 2022), and hamsters (Chan *et al*. 2020), including neurodegenerative pathology in canines (Kim *et al*. 2023). Spillback from animals to humans has been reported for mink (Oude Munnink *et al*. 2021), deer (Feng *et al*. 2023), and hamsters (Yen *et al*. 2022); thus, SARS-CoV-2 presents serious ‘One Health’ implications.

The broad species tropism of SARS-CoV-2 is attributed primarily to the ability of SARS-CoV-2 to use receptors from multiple species, notably angiotensin-converting enzyme 2 (ACE2). Documented ACE2 usage has been reported for SARS-CoV-2 for various species: rhesus macaques, Mexican free-tailed bats, Chinese horseshoe bats, palm civets, raccoon dogs, ferret badgers, hog badgers, white-tailed deer, mule deer, elk, dogs, cats, mink, rabbit, and pangolins (Zhao *et al*. 2020; Palmer *et al*. 2021; Ren *et al*. 2021; Porter *et al*. 2024). SARS-CoV-2 variants harbouring Spike N501Y, including B.1.1.7, B.1.351, P.1, and P.3, also have the ability to infect cells *via* mouse and rat ACE2 (Shuai *et al*. 2021).

Infection of cells is mediated by the interaction of the viral Spike glycoprotein and ACE2 on cells. Spike, which is exposed on the surface of the virion and composed of S1 and S2 subunits (Kreutzberger *et al*. 2022), binds to ACE2 *via* the receptor binding motif within the receptor binding domain of the S1 subunit. Cleavage by host proteases transmembrane serine protease 2 (TMPRSS2) or cathepsins potentiates Spike rearrangement and fusion of the viral and host membranes either in the endosome or at low pH, at the surface of the cell (Wang *et al*. 2021a). The positive-sense viral RNA is released into the cell to initiate translation of the viral genome and subsequent replication.

Replication in animals has been well-characterized for cats: SARS-CoV-2 replicates in the lungs, upper respiratory tract, soft palates, tonsils, distal trachea, tracheobronchial lymph node, small intestines, and kidneys (Shi *et al*. 2020; Rudd *et al*. 2021). Shedding of virus in respiratory droplets leads to transmission between cats (Shi *et al*. 2020). SARS-CoV-2 is also transmitted between white-tailed deer (Palmer *et al*. 2021; Cool *et al*. 2022). In experimental infections of deer, viral RNA has been detected in the nose, mouth, rectum, tonsils, lymph nodes, spleen, liver, kidney, bone marrow, stomach, ileocecal junction, olfactory bulb, heart, brain, cerebrospinal fluid, and large and small intestines. Infectious virus was detected in the trachea, bronchi, nasal wash, and bronchoalveolar lavage fluid (Cool *et al*. 2022).

Several animals susceptible to SARS-CoV-2 and to intra-species transmission frequently interact with humans. Studies suggesting that the frequency of SARS-CoV-2 spillover from humans to deer was previously underestimated after examining phylogenetic relationships between human- and deer-derived SARS-CoV-2 sequences (McBride *et al*. 2023), highlighting the inherent risk of transmission in both directions across the animal–human interface. Mutations in receptor binding proteins often mediate host tropism and can be responsible for increased transmission (Li *et al*. 2005; Hou *et al*. 2020; Kang *et al*. 2021; Cereghino *et al*. 2024). In fact, several mutations in Spike have facilitated increased adaptation to mink (Zhou *et al*. 2022), and there is some evidence for adaptation to deer with a mutation in the ORF3a gene (McBride *et al*. 2023). Therefore, we sought to identify mutations that adapt SARS-CoV-2 to animals, as these could lead to the persistence of SARS-CoV-2 in animal populations. We serially passaged SARS-CoV-2 in cells expressing dog, cat, mink, and white-tailed deer ACE2 and searched for mutations congruent with epidemiologically relevant residues. We identified Spike A222V, which arose in cells expressing cat ACE2, and is a recurrent mutation across eight independent SARS-CoV-2 lineages in human-derived sequences and occurs in animal sequences. Since Spike A222V is also present in animal-derived SARS-CoV-2 sequences, we hypothesized that Spike A222V could be both human and animal adaptive. Thus, we sought to determine the impact of Spike A222V on replication of SARS-CoV-2 in a relevant human cell line and primary deer lung cells and infection *via* the ACE2 receptor of various relevant animal species.

## Materials and methods

### Phylogeny

The phylogenetic tree of SARS-CoV-2 sequences was built by NextStrain (Hadfield *et al*. 2018). The nCoV dataset from GISAID on the NextStrain app was accessed on 19 June 2024. The interactive display was used to highlight the amino acid identity at Spike 222 in ~4000 global sequences with dates from December 2019 to June 2024. Four thousand sequences were selectively displayed by NextStrain for maximum build performance and legibility in the app with automatic, representative subsampling. Wuhan-Hu-1/201 is used as a reference for site numbering, and the tree is rooted using early sequences from Wuhan. An assumed rate of 8 × 10^−4^ nucleotide substitutions per site per year was used.

### Variant analysis from SARS-CoV-2 sequences

Complete SARS-CoV-2 genomes from multiple hosts (including human, dog, cat, mink, and deer) were downloaded from the GISAID EpiCov database (www.gisaid.org) as of 20 June 2024. Genomes with low coverage (defined as having >5% ambiguous bases, N’s) were excluded from the dataset. Additionally, 80 SARS-CoV-2 genomes from deer, made publicly available by McBride *et al*. (McBride *et al*. 2023), were obtained from GenBank for inclusion in the analysis. All sequences were aligned to the SARS-CoV2 reference (NCBI Reference Sequence/NC_045512.2) using Minimap2 (Li 2018) with default settings except for the use of the parameter ‘-axe asm5’. Sequences with aligned lengths <20 000 bp were excluded from the analysis to ensure adequate coverage for variant calling. Variant extraction was performed using the ‘mpileup’ command in Samtools (Li *et al*. 2009).

### Cell lines and plasmids

Baby hamster kidney cells (BHK-21 [C-13]) and human lung epithelial cells (Calu-3 HTB-55) were acquired from the American Type Culture Collection. African green monkey kidney epithelial cells expressing TMPRSS2 and human ACE2 (Vero E6-TMPRSS2-T2A-hACE2; NR-54970) were acquired from BEI Resources. White-tailed deer primary lung cells were provided by the United States Department of Agriculture (USDA). All cells were grown in carbon dioxide (5%) incubator at 37°C with a humidified atmosphere. Primary deer lung cells were grown in Medium 199 (Gibco 12-340-030) with 10% fetal bovine serum (FBS). All other cells were grown in Dulbecco’s modified Eagle medium (Corning™ 10013CV) supplemented with gentamicin sulfate (0.1%), nonessential amino acids (1×), 4-(2-hydroxyethyl)-1-piperazineethanesulfonic acid (HEPES) (25 mM), and either 5% (Vero E6-TMPRSS2-T2A-hACE2 and BHK-21) or 20% (Calu-3) FBS. Vero E6-TMPRSS2-T2A-hACE2 also required the addition of 0.01 mg/ml puromycin. Expression plasmids encoding the human (pGL113), dog (pGL114), cat (pGL116), mink (pGL271), or white-tailed deer ACE2 (pGL369) sequence were shared by B. Zhou and G. Larson at the Centers for Disease Control and Prevention (CDC).

### Passaging experiments and sequencing

BHK-21 cells were transfected with 0.5 μg of either pUC19 or pGL113 (human), pGL114 (dog), pGL116 (cat), pGL271 (mink), or pGL369 (white-tailed deer) expression plasmids for ACE2. Cells were infected in quadruplicate at a multiplicity of infection (MOI) of 0.01 24 h later with SARS-CoV-2 Wuhan-Hu-1 (GenBank accession NC_045512.2) bearing the Spike D614G mutation generated from an infectious clone detailed below. Supernatants were harvested from each replicate at peak titre and quantified by plaque assay on Vero E6 hACE2-TMPRSS2 cells. Supernatants were passaged independently for a total of 10 passages. RNA was extracted from the unpassaged virus and from passage 5 and 10 viruses using the Zymo Research *Quick*-RNA Viral kit (R1035). Library preparation was performed using an Illumina COVIDseq assay kit with ARTIC V3 primers. Paired-end 2 × 150bp sequencing was performed on the Illumina HiSeq platform. Sequencing files were trimmed with bbduk to remove low-quality reads and ARTIC primer sequences.

### Generation of live SARS-CoV-2 mutants

Mutant viruses were generated using a full-length infectious cDNA clone of the Wuhan-Hu-1 strain of SARS-CoV-2 (GenBank accession no. NC_045512.2). Mutagenic polymerase chain reactions (PCR) were performed to generate overlapping PCR fragments from the clone using Invitrogen Platinum SuperFi II PCR master mix (12368010) or Quantabio repliQa HiFi ToughMix (95200-025). PCR fragments were digested to cut residual PCR template and gel purified using the Machery-Nagel nucleospin gel and PCR clean-up kit (740609.250). Purified fragments were assembled using the OriCiro Genomics 2× RA master mix. Unassembled product was digested, and the assembled plasmid containing the OriC cassette was amplified by replication cycle reaction using the OriCiro Genomics 10× RE mix. Virus was rescued in a BSL-3 laboratory by DNA transfection of BHK-21 cells using a 1:1 mix of amplified replication cycle reaction product and pUC19 with the Polyplus jetOPTIMUS DNA transfection reagent (101000051). At 2 and 3 days post-transfection, supernatant was blind passaged on Vero E6-TMPRSS2-T2A-ACE2 cells, and virus was harvested at 25% cytopathic effect. Viral genomic sequences were validated by preparing libraries with IDT Artic V4.1 NCOV-2019 Panel (10011442) and were sequenced using Genewiz NGS Amplicon-EZ services with 2 × 250 bp reads.

### Growth curves and infections with transient ACE2 expression

All live virus manipulation was performed at BSL-3. Human lung epithelial cells (Calu-3) and baby hamster kidney cells (BHK-21) were infected at 80% confluency with an MOI of 0.1. White-tailed deer primary lung cells were infected at an MOI of 1. All cells were infected with either SARS-CoV-2 Wuhan-Hu-1 with the Spike D614G mutation (wild-type) or SARS-CoV-2 Spike A222V-D614G diluted in Roswell Park Memorial Institute 1640 (RPMI-1640) medium supplemented with FBS (2%) and HEPES (10 mM). Supernatant was harvested every 24 h postinfection and titred by plaque assays on Vero E6-TMPRSS2-T2A-hACE2 cells.

### Titration of infectious virus

Infectious virus was quantified by plaque assay by infecting 90% confluent monolayers of Vero E6-TMPRSS2-T2A-hACE2 with serial dilutions of virus made in RPMI-1640 medium supplemented with FBS (2%) and HEPES (10 mM). Adsorption was performed for 1 h at 37°C. Following adsorption, a 1:1 mixture of 2× media (2× Eagle's Minimum Essential Medium, 4× L-glutamine, 0.735% sodium bicarbonate, 0.2 mg/ml gentamicin sulfate, 4% FBS, 20 mM HEPES) and 3% methylcellulose was added to the cells. Cells were fixed after three days of infection using a 10% buffered formalin solution. Cells were stained with a 0.1% crystal violet solution containing 20% ethanol to visualize plaques.

## Results

### Passaging of SARS-CoV-2 in cells expressing animal ACE2 results in minimal high-frequency spike mutations

**Figure 1:**
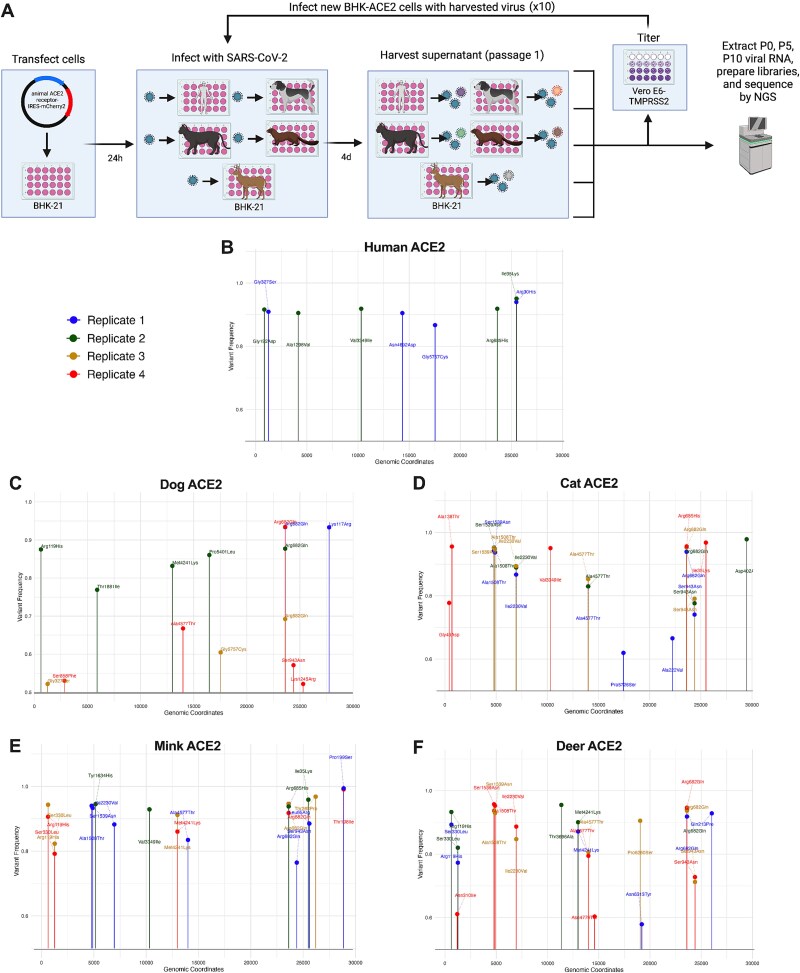
Mutations in SARS-CoV-2 following passaging in cells expressing animal ACE2. The Wuhan-Hu-1 strain of SARS-CoV-2 with the Spike D614G mutation was passaged 10 times at an MOI of 0.01 in BHK-21 cells expressing a human, dog, cat, mink, or white-tailed deer ACE2 receptor. All passaging was performed in four replicates, except for human ACE2, which had two replicates. Passaged populations were sequenced *via* next-generation sequencing, and mutations are reported across the length of the genome. Variants were called with a *P*-value threshold of .01 after Bonferroni correction, and only those which exceeded 0.5 variant frequency are shown. (A) Schematic of passaging experiment. (B–F) SARS-CoV-2 mutations after 10 passages in BHK-21 cells expressing human (B), dog (C), cat (D), mink (E), or white-tailed deer (F) ACE2. Figure 1A created in BioRender with CC-BY-4.0 license. Weger, J. (2025) https://BioRender.com/94xs1i4

SARS-CoV-2 is described as a generalist virus (Tan *et al*. 2022) because of its ability to use several species as hosts (Abdel and Abdelwhab 2020; Conceicao *et al*. 2020). Despite this generalist nature, several animal-adaptive mutations have been identified (Zhou *et al*. 2022), raising concerns about how these changes may affect the transmission dynamics, virulence, and immune evasion of SARS-CoV-2. In addition, the evolutionary rates of SARS-CoV-2 are elevated in animals like farmed mink (Porter *et al*. 2023), white-tailed deer (McBride *et al*. 2023), and cats (Bashor *et al*. 2022), suggesting that animal-adaptive mutations could arise in these animals and lead to the establishment of new animal reservoirs. To determine whether animal-adaptive mutations would arise after repeated exposure to animal ACE2, we serially passaged SARS-CoV-2 in a cell culture model of transient animal-ACE2 expression. Baby hamster kidney cells (BHK-21), a permissive but nonsusceptible cell line (Supplementary Fig. 1), transiently expressing either dog, cat, mink, deer, or human ACE2 were infected with the Wuhan-Hu-1 strain of SARS-CoV-2 bearing the Spike D614G, a likely human-adaptive mutation present in nearly all sequences after late 2020 (Plante *et al*. 2021). We performed 10 passages at an MOI of 0.01 and sequenced the input virus and the populations following 5 (p5) and 10 (p10) passages (Fig. 1A).

We observed consensus-level, nonsynonymous mutations within ORF1a, ORF1b, Spike, ORF3a, ORF7a, and nucleocapsid of the passaged populations in all ACE2 environments (Fig. 1B–F); however, we only discuss those found in Spike given the well-characterized interaction between Spike and ACE2. All mutations, including those outside of Spike, are detailed in supplementary file 1. Spike R685H was observed at p10 in one replicate (1/2) of cells expressing human ACE2 and in p5 and p10 for one replicate (1/4) of cells expressing cat ACE2 in addition to mink ACE2 (1/4) (Fig. 1B, D, and  E). Spike R682Q arose in both p5 and p10 for all replicates of dog ACE2 (4/4), three of cat ACE2 (3/4), three of mink ACE2 (3/4), and all of deer ACE2 (4/4) but not human ACE2 (0/2) (Fig. 1B–F). Previous studies have shown that Spike R682Q and R685H arise during passaging in TMPRSS2-deficient Vero cells (Ogando *et al*. 2020; Sasaki *et al*. 2021) and, consequently, are likely adaptations towards the BHK-21 cells we used. Spike S943N arose at both p5 and p10 in one replicate (1/4) of cells expressing dog ACE2, three replicates (3/4) of cells expressing cat ACE2, one replicate (1/4) of cells expressing mink ACE2, and two replicates (2/4) of cells expressing deer ACE2 but not in cells expressing human ACE2 (Fig. 1B–F). Finally, Spike A222V, which lies in the receptor binding domain (RBD), arose in both p5 and p10 in one replicate (1/4) of cells expressing cat ACE2 (Fig. 1D). This mutation was characteristic of the 20E (EU1) variant and later the Delta subvariant AY.4.2 (Ginex *et al*. 2022). Given that Spike A222V and Spike S943N arose in both p5 and p10 of cells expressing animal ACE2, we posited that these mutations may confer an adaptive advantage for SARS-CoV-2 through infection *via* animal ACE2.

### Prevalence of mutations from passaging experiments in SARS-CoV-2 sequences from humans and animals across phylogeny and time

**Figure 2:**
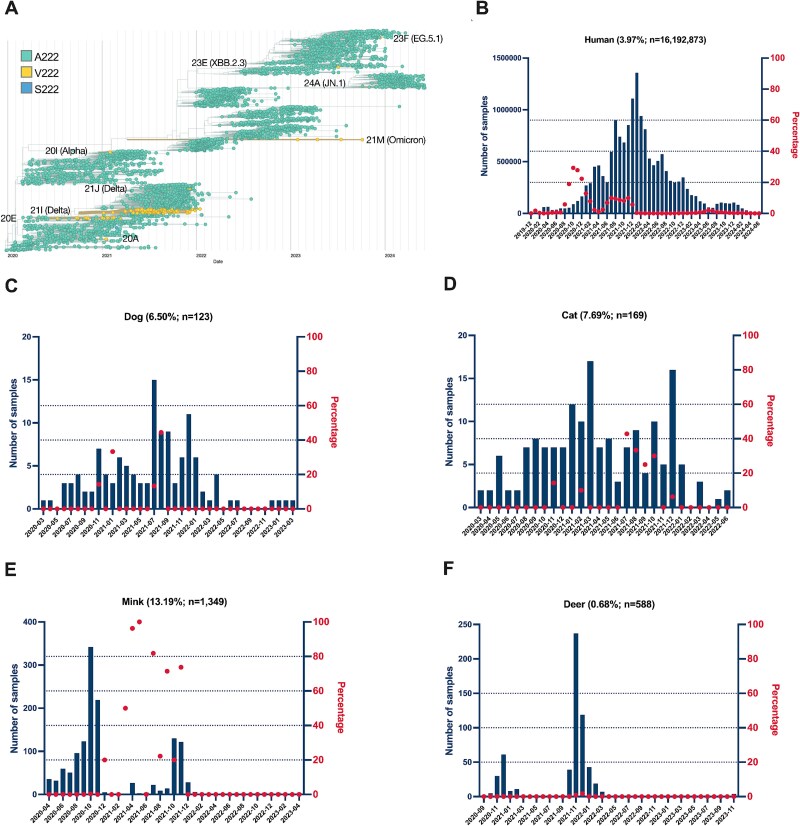
Spike A222V reoccurs in human and animal sequences across time and clade. (A) Phylogenetic tree of SARS-CoV-2 sequences from all hosts on GISAID. Phylogeny represents ~4000 genomes selected by NextStrain. The amino acid identity Spike V222 is highlighted in the tree (yellow). Clades are annotated according to sequences containing Spike V222. (B–F) Frequency of the Spike A222V mutation in SARS-CoV-2 sequences found in different hosts over time. Frequencies are shown for humans (B), dog (C), cat (D), mink (E), and white-tailed deer (F). The bars represent the number of samples collected in each month. The dots indicate the percentage of samples with the A222V mutation in a given month. The percentage within parentheses following the name of each host for each panel [e.g. Human (3.97%)] denotes the overall frequency of the A222V mutation observed in that host. Phylogenetic tree was generated by NextStrain and is used under a CC-BY-4.0 license.

Animal-adaptive SARS-CoV-2 mutations have been associated with outbreaks and mortality in farmed animals and have spilt back to humans, thus classifying such mutations as critical One Health threats. To identify animal-adaptive mutations in SARS-CoV-2 that constitute important targets in a One Health framework, we searched for Spike mutations A222V and S943N identified from our passaging experiments among other human- and animal-derived SARS-CoV-2 sequences. While Spike S943N arose in seven passaging replicates, this mutation was not detected in any circulating SARS-CoV-2 sequences and thus is likely not relevant to infection of a natural host. In contrast, Spike A222V was a recurring mutation in human-derived SARS-CoV-2 sequences from distinct clades as previously reported (Ginex *et al*. 2022) (Fig. 2A). The natural reoccurrence of Spike A222V in human-derived SARS-CoV-2 sequences marked its relevance to public health, and as such, we investigated this mutation further to determine why it may recur. Spike A222V is a mutation in the N-terminal domain of SARS-CoV-2 that shows signals of positive selection and was associated with both the 20EU and Delta variants (Ginex *et al*. 2022). Spike A222V is detected sporadically and to very low frequencies in more recent lineages, some of which include Omicon (21M), JN.1, XBB.2.3, and EG.5 (Fig. 2A). We detected Spike A222V in 642 857 of 16 192 873 (3.97%) human-derived SARS-CoV-2 sequences from GISAID (Fig. 2B). We next asked whether dog, cat, mink, or white-tailed deer SARS-CoV-2 sequences also harbour Spike A222V since these animals closely interact with humans and support SARS-CoV-2 transmission (Bosco-Lauth *et al*. 2020; Oude Munnink *et al*. 2021; Lyoo *et al*. 2023; McBride *et al*. 2023). Spike A222V is present in 6.5% (8/123) of dog-derived sequences, 7.69% (13/169) of cat-derived sequences, 13.19% (178/1349) of mink-derived sequences, and 0.68% (4/588) of white-tailed deer-derived sequences (Fig. 2C–F). Based on the presence of Spike A222V in these sequences, we hypothesized that Spike A222V could be adaptive across a wide range of species, including humans.

### SARS-CoV-2 Spike A222V replicates to higher levels in primary deer lung epithelial cells but has no effect on replication in human lung epithelial cells

**Figure 3:**
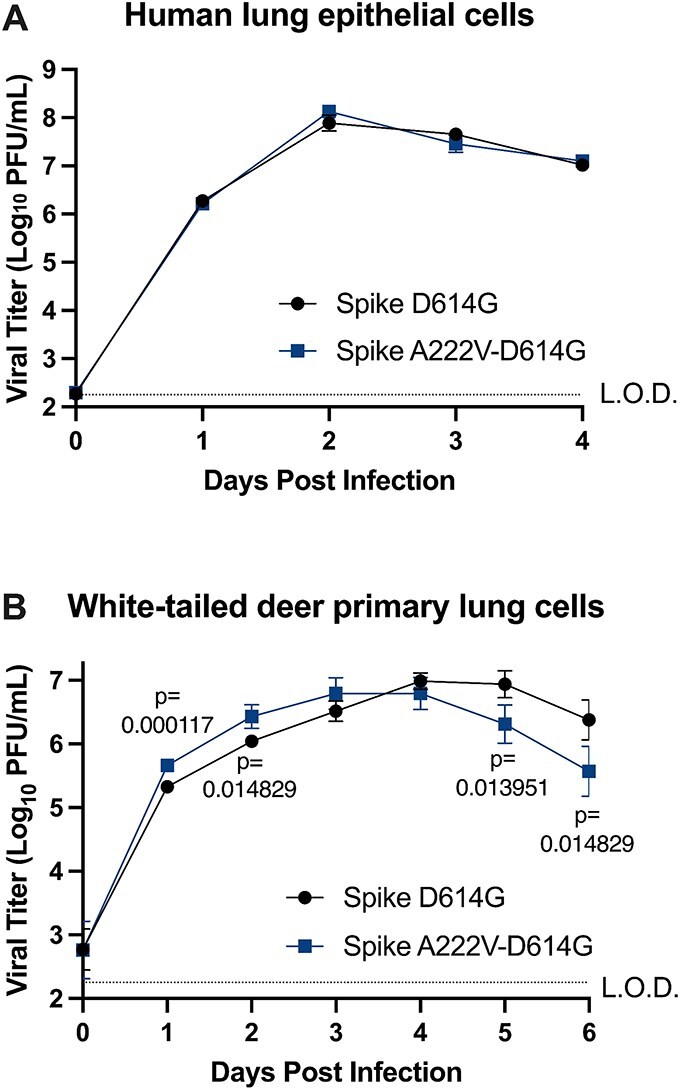
Replication of SARS-CoV-2 Spike A222V in human and deer lung cells. (A) Replication of SARS-CoV-2 Spike A222V-D614G in human lung epithelial cells (Calu-3). Cells were infected with an MOI of 0.1, and supernatant was collected each day postinfection. Infectious virus was titrated on VeroE6-human ACE2-TMPRSS2 cells. Means represent the average of six technical replicates performed in two independent biological replicates. (B) Replication of SARS-CoV-2 Spike A222V-D614G in white-tailed deer primary lung cells. Cells were infected with an MOI of 1, and supernatant was collected each day postinfection. Infectious virus was titrated on VeroE6-hACE2-TMPRSS2 cells. Means represent the average of six technical replicates. Growth curve data (A and B) are representative of two independent biological replicates. Statistical comparisons were made using multiple unpaired *t*-tests with Welch correction and Holm–Šídák’s correction for multiple comparisons where α = 0.05. Error bars represent standard deviation. L.O.D., limit of detection.

Due to the prevalence of Spike A222V in both human and animal-derived SARS-CoV-2 sequences, we first asked whether Spike A222V confers an advantage for SARS-CoV-2 during replication in human cells. Previous biochemical studies examining the binding affinities of Spike A222V-D614G and Spike D614G to human ACE2 (hACE2) determined that Spike A222V-D614G binds to hACE2 with a higher affinity (Ginex *et al*. 2022), suggesting that this mutation could provide an advantage during entry into human cells. To determine if there are differences in replication kinetics of SARS-CoV-2 bearing Spike A222V in human cells, we constructed a live, Spike A222V virus in the Wuhan-Hu-1 background with Spike D614G. We infected human lung epithelial cells (Calu-3) with SARS-CoV-2 Spike D614G and Spike A222V-D614G at an MOI of 0.1. Notably, SARS-CoV-2 Spike A222V-D614G did not replicate differently from SARS-CoV-2 Spike D614G alone (Fig. 3A).

While Spike A222V has a neutral effect on the replication of SARS-CoV-2 in human lung cells, we sought to test the hypothesis that Spike A222V is animal-adaptive. While we could not obtain primary cat, dog, or mink cells to infect with SARS-CoV-2 despite the higher frequency of Spike A222V in SARS-CoV-2 sequences from these host species, we instead infected primary deer cells. Deer are a highly relevant species given their ability to transmit SARS-CoV-2 to other deer in nature with potential spillover to humans (Feng *et al*. 2023), and they show evidence of evolution of SARS-CoV-2 (McBride *et al*. 2023) and constitute an important One Health host for their interaction with many species, including humans. We posited evolution of SARS-CoV-2 in deer and their maintenance of the virus could be likely and thus sought to test whether Spike A222V could increase SARS-CoV-2 replication in primary deer cells if it were to arise *de novo* in deer or spillover from other species. We infected white-tailed deer primary lung cells with SARS-CoV-2 Spike A222V-D614G and Spike D614G at an MOI of 1. SARS-CoV-2 Spike A222V-D614G replicated to significantly higher titres earlier than Spike D614G (Fig. 3B). Thus, Spike A222V increases fitness of SARS-CoV-2 in primary deer lung epithelial cells.

### Spike A222V does not enhance SARS-CoV-2 replication in deer cells *via* the ACE2 receptor

**Figure 4:**
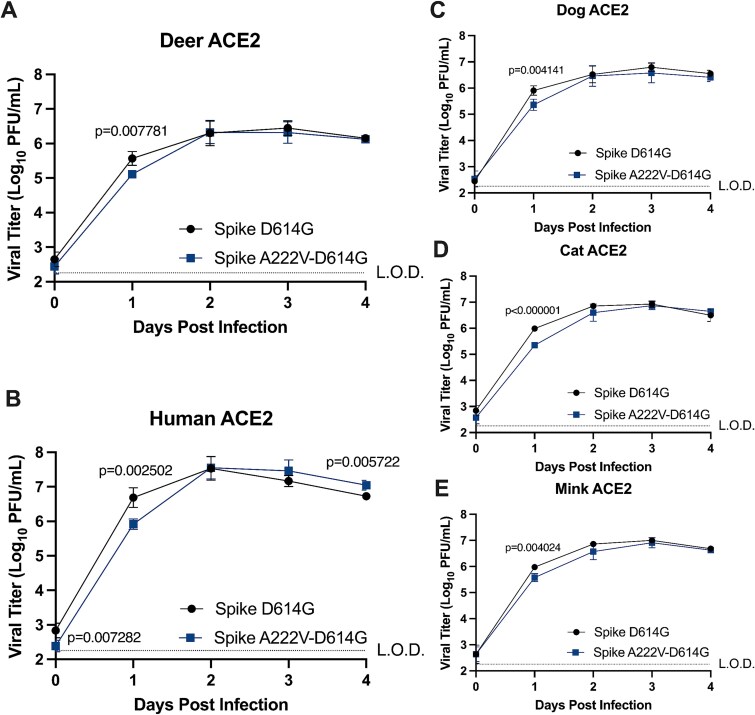
Replicative fitness of Spike mutants with cells expressing deer, human, dog, cat, and mink ACE2. (a–e) Replication of SARS-CoV-2 Spike A222V-D614G in BHK-21 cells expressing animal ACE2 receptors. BHK-21 cells were transfected with expression constructs for deer (A), human (B), dog (C), cat (D), or mink (E) ACE2. Cells were infected 24 h later at an MOI of 0.1, and supernatant was collected each day postinfection. Infectious virus was titrated on VeroE6-human ACE2-TMPRSS2 cells. Data comprise two independent biological replicates, and means represent the average of six technical replicates. Statistical comparisons were made using multiple unpaired *t*-tests with Welch correction and Holm–Šídák’s correction for multiple comparisons where α = 0.05. Bars represent the standard deviation of the mean. L.O.D., limit of detection.

Since Spike A222V is near the receptor binding domain of Spike, we hypothesized that increased replication in deer cells was mediated by increased entry *via* deer ACE2. To test this, we inoculated cells transiently expressing different animal ACE2 receptors with either SARS-CoV-2 Spike A222V-D614G or Spike D614G at an MOI of 0.1. Spike A222V-D614G displayed reduced titres compared to Spike D614G in BHK-21 cells expressing deer ACE2 (Fig. 4A). Replication was also diminished *via* human, dog, cat, and mink ACE2 (Fig. 4B–E) at 1 day postinfection. Replication of SARS-CoV-2 Spike A222V-D614G returned to titres similar to Spike D614G by 2 days postinfection in all cells. These data suggest SARS-CoV-2 replication is attenuated by Spike A222V at the step of entry, though only when entry occurs *via* ACE2. In combination with the positive impact on replication in primary deer lung cells, these data reveal the complex role Spike A222V plays in entry *via* ACE2-dependent and independent host factors.

## Discussion

SARS-CoV-2 mutations that confer replication advantages in animals have resulted in large animal outbreaks, resulting in animal mortality (Oreshkova *et al*. 2020), economic losses to farming industries (Wang *et al*. 2021b), and spillover events to humans (Oude Munnink *et al*. 2021). With an aim to identify key animal-adaptive mutations, we experimentally evolved SARS-CoV-2 in the presence of cells expressing animal ACE2. We identified a mutation, Spike A222V, from the passaging experiment that recurred in human-derived sequences and was also present in dog-, cat-, mink-, and deer-derived SARS-CoV-2 sequences. We assessed the replicative fitness of Spike A222V in the Wuhan-Hu-1 background with Spike D614G and determined that it replicates to higher levels in primary deer lung epithelial cells.

The Spike A222V mutation in SARS-CoV-2 recurred in at least eight different lineages of human-derived SARS-CoV-2 sequences and was present in animal sequences. Biochemical and structural simulations have characterized the effect of the Spike A222V mutation on Spike stability and potential human ACE2 receptor binding (Ginex *et al*. 2022), but our study is the first to examine the effect of Spike A222V on replication of authentic SARS-CoV-2 *in vitro* and to test replication in human and animal cells. Based on an increase in affinity of Spike A222V for human ACE2 (Ginex *et al*. 2022), we hypothesized that Spike A222V would increase the replication of SARS-CoV-2 in human cells *via* enhancing interaction with the ACE2 receptor. We infected cells transiently expressing human ACE2 that were otherwise not susceptible to infection and surprisingly observed decreased replication of the mutant bearing Spike A222V. Furthermore, we found no difference in the replication of Spike A222V-D614G and Spike D614G viruses in the Wuhan-Hu-1 background in human lung epithelial cells (Calu-3). Since Spike A222V first arose in the 20E (EU1) variant background and not the Wuhan strain that we use here, it is possible that other mutations characteristic of the 20E (EU1) variant (ORF10 V30L, N A220V, and ORF14 L67F) work synergistically with Spike A222V. The importance of epistatic interactions with Spike A222V has been demonstrated by others and could explain the recurring nature of Spike A222V in human-derived SARS-CoV-2 sequences where the mutation enhances the fitness of the virus only in certain backgrounds. Spike A222V also negatively impacted replication in cells expressing cat ACE2, though this mutation arose in this same environment during passaging. Since our passaging experiments did not impose a significant bottleneck on the viral populations (1 virus particle per 100 cells), it is most likely that Spike A222V confers an advantage in replication *via* cat ACE2 only in the context of the other Spike mutations we identified: R682Q and S943N. While we did not construct and examine a mutant bearing all of these Spike mutations, we can speculate that these may have been required for Spike A222V to arise to such a high frequency. While we did not determine that Spike A222V enhances the fitness of SARS-CoV-2 in cells that express human or cat ACE2, this may not be true with other variant backgrounds. Future studies should examine this interplay of mutations *in vitro*.

SARS-CoV-2 is classified as a generalist virus that has seldom evolved by positive selection in animals. Instead, the majority of deterministic processes of evolution have been caused by purifying selection and stochastic processes (Braun *et al*. 2021; McBride *et al*. 2023). However, a few instances of adaptation to animals *via* positive selection have been documented. The cluster 5 variant of SARS-CoV-2, which caused an outbreak on mink farms in 2020, harboured the Spike Y453F mutation that enhanced interaction of Spike with mink ACE2 (Ren *et al*. 2021). Experimental infection of ferrets with SARS-CoV-2 also resulted in mutation to Spike 453F and Spike 501T, which were associated with increased viral shedding in ferrets (Zhou *et al*. 2022). Spike F485L, which has arisen multiple times in mink-derived SARS-CoV-2 sequences, adapts Spike to enhanced entry *via* ferret ACE2 (Zhou *et al*. 2022). At least 58 sites under positive selection have been detected in SARS-CoV-2 from spillover events from humans to deer (Feng *et al*. 2023), and Spike H655Y was rapidly fixed in experimental inoculation of cats (Braun *et al*. 2021), though none of these mutations have been validated experimentally. In the present study, we identified a mutation in Spike at amino acid 222 that is a putative deer-adaptive mutation. While <1% of deer-derived SARS-CoV-2 sequences harbour this mutation, it is possible that the mutation is underrepresented as no deer sequences were available beyond the beginning of 2022. Live transmission studies in deer could elucidate whether this mutation arises *de novo* and whether it impacts deer-to-deer transmission or pathogenesis.

Spike A222V was represented at the highest frequency in mink-derived sequences (13%) but, surprisingly, showed decreased replication in cells expressing mink ACE2. We saw a similar result with human, dog, cat, and deer ACE2. Spike A222V attenuated SARS-CoV-2 replication *via* deer ACE2, yet in primary lung cells from white-tailed deer, replicative fitness of SARS-CoV-2 was enhanced by Spike A222V. A similar result was obtained in Calu-3 cells, where the Spike A222V showed equivalent replication to the WT virus, but in BHK-21 cells expressing human ACE2, the mutant was attenuated. This suggests that other entry or attachment factors present on primary deer lung cells or Calu-3 cells could be interacting differentially with Spike V222 compared to Spike A222. TMPRSS2 is highly expressed in Calu-3 cells (Koch *et al*. 2021) and is known to increase the infectivity of SARS-CoV-2 (Zang *et al*. 2020). While studies have not validated biochemically whether BHK-21 cells express this protease, the lungs of white-tailed deer express TMPRSS2 (Martins *et al*. 2022). The presence or absence of TMPRSS2 and differential dependence of Spike on TMPRSS2 could be responsible for altering the replication of the Spike A222V mutant in Calu-3 and deer lung cells *versus* BHK-21 cells expressing the ACE2 receptor. It would be useful to determine if Spike A222V has a positive impact on replication and which host factors are responsible for this in other susceptible animal cell lines, tissues, or during live transmission studies since we were limited by cell availability and animals for transmission experiments.

Our approach of serial passaging identified Spike A222V as a naturally recurring mutation that enhances replication in deer lung cells. While further examination of the infection, pathogenicity, and transmissibility of SARS-CoV-2 bearing Spike A222V to and between deer should be performed, surveillance efforts in animals should continue to monitor for this mutation and other putatively animal-adaptive mutations that could be under positive selection.

## Supplementary Material

Supplementary_Figure_1_caption_veaf059

Supplementary_figure_1_veaf059

Supplementary_File_NextClade_Report_veaf059

## Data Availability

The sequencing data have been deposited in the NCBI Sequence Read Archive (SRA) under BioProject accession number PRJNA1291747.
