# Supplementary material for: The recurrent Spike A222V mutation in SARS-CoV-2 enhances replication in primary deer lung cells
Source: Virus Evol. 2025 Aug 5;11(1):veaf059. doi: 10.1093/ve/veaf059 (PMC12378747; doi:10.1093/ve/veaf059)
Supplement: Supplementary_Figure_1_caption_veaf059 [file supplementary_figure_1_caption_veaf059.docx]

**Supplementary Figure 1.** Growth curve of SARS-CoV-2 Spike D614G in BHK-21 cells expressing dog, cat, mink, deer, or human ACE2. BHK-21 cells were transfected with expression constructs for human, dog, cat, mink, or deer ACE2 with pUC19 as a control. Cells were infected at a MOI of 0.1, and supernatant was harvested every 24h and titered by plaque assay. Data represent four technical replicates. L.O.D.=limit of detection.
