# Supplementary figures and images for: The recurrent Spike A222V mutation in SARS-CoV-2 enhances replication in primary deer lung cells
Source: Virus Evol. 2025 Aug 5;11(1):veaf059. doi: 10.1093/ve/veaf059 (PMC12378747; doi:10.1093/ve/veaf059)

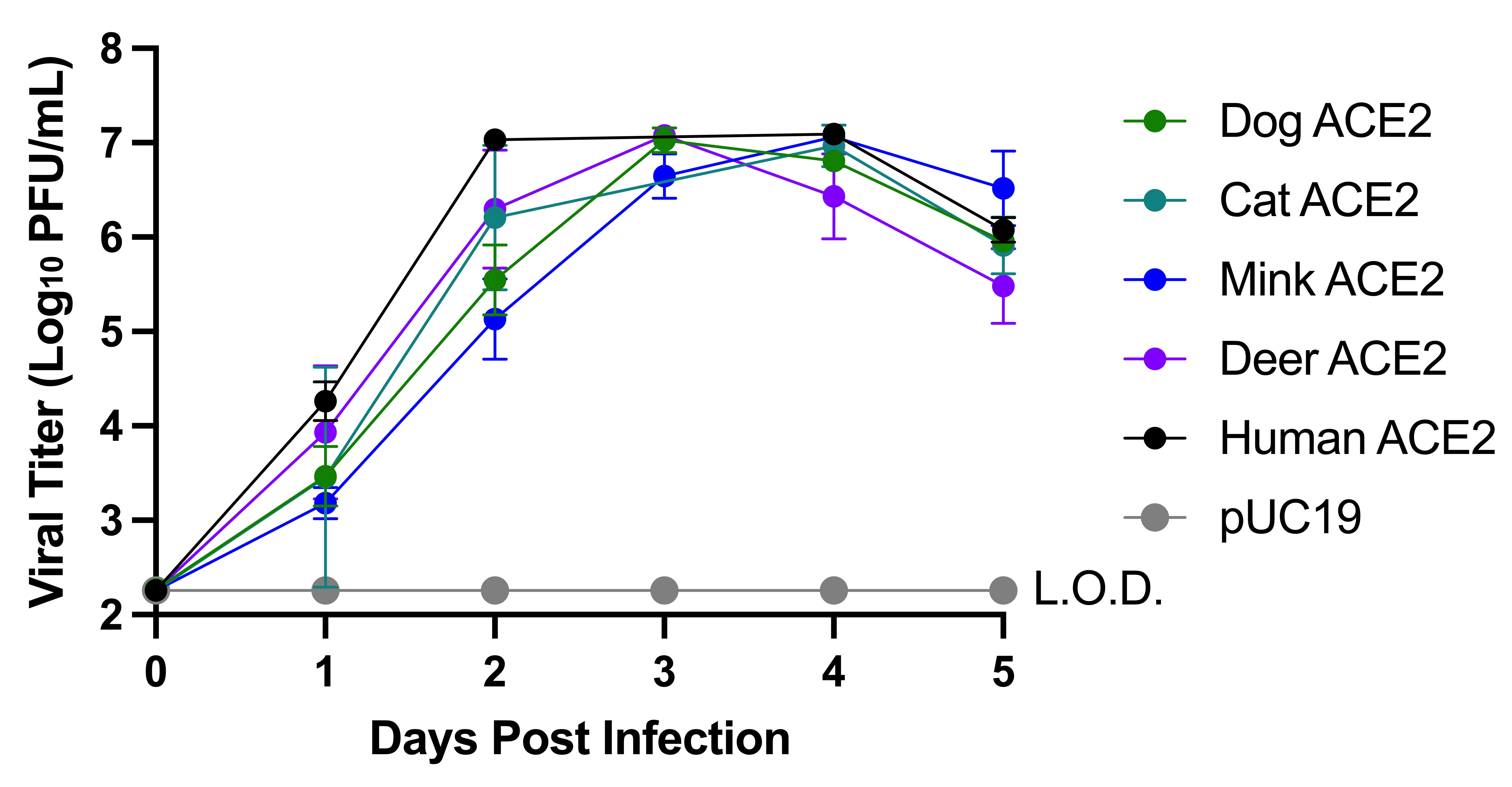

Supplement: Supplementary_figure_1_veaf059 [file supplementary_figure_1_veaf059.jpeg]
